# Mapping Interdisciplinary Fields: Efficiencies, Gaps and Redundancies in HIV/AIDS Research

**DOI:** 10.1371/journal.pone.0115092

**Published:** 2014-12-15

**Authors:** Jimi Adams, Ryan Light

**Affiliations:** 1 Department of Health and Behavioral Sciences, University of Colorado Denver, Denver, Colorado, United States of America; 2 Department of Sociology, University of Oregon, Eugene, Oregon, United States of America; Katholieke Universiteit Leuven, Belgium

## Abstract

While interdisciplinarity continues to increase in popularity among funders and other scientific organizations, its potential to promote scientific advances remains under-examined. For HIV/AIDS research, we examine the dynamics of disciplinary integration (or lack thereof) providing insight into a field's knowledge base and those questions that remain unresolved. Drawing on the complete histories of two interdisciplinary journals, we construct bibliographic coupling networks based on overlapping citations to identify segregation into research clusters and estimate topic models of research content. We then compare how readily those bibliographic coupling clusters account for the structuring of topics covered within the field as it evolves over two decades. These comparisons challenge one-dimensional and/or cross-sectional approaches to interdisciplinarity. Some topics are increasingly coordinated across disciplinary boundaries (e.g., vaccine development); others remain relatively segmented into disconnected disciplinary domains for the full period (e.g., drug resistance). This divergence indicates heterogeneity in interdisciplinarity and emphasizes the need for critical approaches to studying the organization of science.

## Introduction

Many contemporary global problems transcend disciplinary boundaries imposed by institutions of higher education. Solutions to problems such as poverty, conflict, resource depletion, or disease likely demand interdisciplinary collaboration. HIV/AIDS research, for example, does not belong to any single discipline, with scientists making important contributions from a range of basic, clinical, social and applied sciences [1]. Moreover, recent initiatives from funders and other institutional actors have prioritized increasing interdisciplinary integration [2], [3]. In fact, HIV/AIDS research could be considered innovative in this trend having separate interdisciplinary review panels from early in the life-course of NIH funding for HIV/AIDS research [1]. These integrative aims presuppose that research connecting diverse perspectives will more rapidly produce solutions to the most pressing scientific and public health issues [3]–[5]. Despite these lofty goals, the utility of interdisciplinary research is difficult to evaluate [6], [7].

The stakes of interdisciplinarity remain high because poor coordination could limit the potential for important scientific advances. Additionally, large societal investment is banking on the success of these approaches. In socio-medical research, successful communication can result in strategies to improve population health outcomes [8]–[10], for example the discovery and implementation of tactics for preventing mother to child transmission of HIV. Conversely, its absence may partially account for insufficient progress towards other goals, such as the large numbers of people currently living with HIV/AIDS who continue to lack access to appropriate treatment regimens. At the same time, interdisciplinarity may siphon funding and attention away from research questions that demand more focused, disciplinary research. How do we account for the promises and pitfalls of interdisciplinary research?

Scholars studying the structure of scientific production have long-recognized the importance of informal interactions, including citation practices, which bridge traditional disciplinary boundaries for shaping the content and progress of fields [11]. Moreover, the ways these interactions cross disciplinary boundaries can help to shape what is known and how scientists evaluate what questions are worth addressing and what evidence “counts” when providing answers [12], [13]. Work that bridges disciplinary boundaries can take many forms, each having differing implications for how problems get addressed [14]. At the extremes, disciplinarity constrains topics within single disciplinary boundaries, and transdisciplinarity eliminates the salience of disciplinary boundaries altogether. Most integrative work exists somewhere in between; a field organized in an “interdisciplinary” fashion is marked by literatures that combine ideas across disciplinary boundaries to jointly address topic-based research problems [3]. “Multidisciplinary” research incorporates broad simultaneous engagement with research questions that incorporates many disciplinary perspectives, but does so in a way that retains disciplinary separation [3]. Moreover, evaluating how open or resolved questions in a field compare/differ in their respective trajectories across these forms can help to identify not just if, but *how* integrative efforts in problem-based areas of science successfully navigate these processes of disciplinary integration.

Recent work demonstrates the utility of scientometric approaches for accounting for boundary structure and dynamics to examine the whole of science [4], [15], or for single academic disciplines [16], [17]. These approaches provide tools that are well suited to address questions of interdisciplinary integration in research fields like HIV/AIDS [18], [19]. These tools can help us identify cross-sectional patterns within scientific communities and can explicate how those patterns evolve over the life course of fields [20]. As such, we examine how integrated the field of HIV/AIDS research was over a two decade period and how that integration evolved as the field matured. We discuss the implications of that structuring as it accounts for particular scientific discoveries (e.g., the development and implementation of antiretroviral therapies) and characteristic areas that remain unresolved.

## Data and Analyses

Our data come from all published articles, letters and notes in the two top interdisciplinary journals for HIV/AIDS research – *AIDS* and *JAIDS* – from their respective first issues through the end of 2008. This includes a total of 16,907 published items (10,218 from *AIDS* and 6,689 from *JAIDS*). We retrieved the full bibliographic information (including complete cited references lists) and abstract text for each of these items from ISI Web of Science. Analyses address this complete corpus and each journal separately. To identify the structure and content of research communities in the *AIDS/JAIDS* corpus, we combine bibliographic coupling networks with topic models, presenting results for the complete time-collapsed corpus (i.e., treating the full corpus as a single literature) and a series of time-based moving windows to examine the evolution of patterns over two decades.

**Figure 1:**
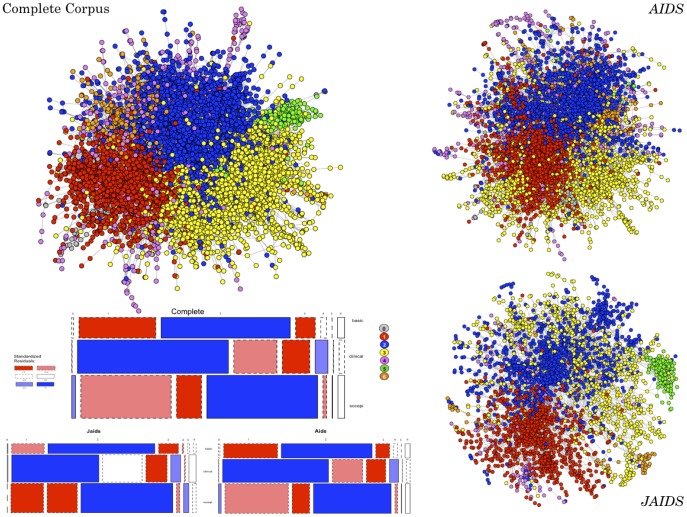
Bibliographic Coupling Network Communities in the Complete Corpus. Panel A presents the full bibliographic coupling network, edge-reduction is based on papers with weighted similarity scores two standard deviations above the median similarity among non-isolates in the network. Node color represents each paper's identified bibliographic coupling community using the Newman-Girvan algorithm [26]. Panels B and C present the same analyses limited only to publications from *AIDS* and *JAIDS* respectively. Panel D show the correspondence between communities and the broad “discipline” like labels applied to all published *articles* beginning in 1998. Color represents whether a label is over (blue) or under (red) represented in a given community according permutation-based residuals.

First, for each pair of papers in the corpus, we construct a paper-to-paper bibliographic coupling network [21], [22]. To construct the bibliographic coupling network, we use data preprocessing capabilities in [23] to compute the extent to which papers in our corpus (N = 16,907) jointly cite the same papers, using cosine-weighted cited-reference similarity scores [24]; results did not differ appreciably when alternatively employing weights based on simple citation counts or Jaccard similarity [25]. All bibliographic-coupling network analyses presented in the paper rely on these fully weighted cited reference similarity scores. However, to reduce some of the noise in visualizations, the network representations in Fig. 1 recode this similarity matrix to dichotomous presence/absence of ties between paper pairs with similarity scores that exceed the mean score plus two standard deviations; this computation excludes all isolates (i.e., those papers that share no citations with any other papers in the corpus).

Second, we analyze those networks with community detection approaches, which identify segmentation within a network [26], [27]. Formally, this is generally computed as locating blocks of the network for which some majority of ties are formed within the group and relatively few ties are formed outside those groups [27]. There are numerous strategies for finding network communities; here we use the fast-greedy algorithm [28] for computing the Newman and Girvan [26] index as implemented in igraph 0.6–2 [29] for R 3.0.1; results did not differ appreciably when using the Louvain method as an alternative [30]. Modularity maximization is a common strategy for finding the number of communities in a graph and can be used to describe how readily the identified communities account for the structure of an observed network [31]. Modularity scores represent locally maximized functions that identify how readily ties form within as opposed to across communities. Our results below rely on solutions that identify between 6–9 communities identified (depending on the period). While the raw interpretation of modularity scores is rare, comparison across networks with similar numbers of nodes and ties can reveal any substantial changes in community structure over time [27], which we summarize by plotting the structural changes over time. We then use an Alluvial Flow diagram described in [32] to visualize how the detected communities change over time.

Third, since the *content* of science is also essential to understanding interdisciplinarity, we generate a topic model for the abstract texts in the corpus. Topic models consist of a class of techniques that locate structure in unstructured text corpora [33], [34]. They “reverse engineer” the writing process to uncover latent themes within the corpus that underlie the generative processes for producing each document [35]. While several alternatives and specifications exist [35], [36], we use latent dirichlet allocation (LDA) as implemented by lda 1.3.2 in R [36]. LDA is a Bayesian approach to modeling language that assumes that texts consist of a distribution of hidden themes or topics. We empirically identify a fixed number of topics (k = 30, see S1 Figure and S1 Table for more details), but the distribution of topics over abstracts is not fixed. A topic consists of a distribution of words, here a dirichlet distribution. LDA presents several advantages over alternatives. First, as a hierarchical model, LDA consists of three levels: the corpus, the document, and the word. Second, and most importantly for our discussion, documents do not have to be assigned to single topics. Operationally, abstracts can be assigned with proportional probabilities to multiple topics [35].

Fourth, we compare how readily these topics are contained within or bridge across the identified bibliographic coupling communities. We do this with residual contingency analyses for categorical independence, which we visualize with mosaic plots [37]. A random distribution of topics over clusters (neither over- *nor* under- representation across clusters) suggests that clustering is not at all topic-related. Underrepresentation alone can help identify topics that are not salient for the development of particular bibliographic coupling clusters, while *consolidation* is marked by topics with high over-representation in *one* cluster and underrepresentation in others. Lastly, those single topics that are over-represented in multiple clusters lack integration in that the same topics are being covered in clusters that are not drawing upon the same literatures to develop ideas within them – i.e., are more multidisciplinarily organized. In combination, these approaches allow us to identify how segmented or consolidated the HIV/AIDS research field is, and how disciplinary boundaries contribute to that structuring, in part by identifying which topics are well-bounded within single research communities versus those that span across several.

Moreover, by examining how this alignment shifts across the observed window, we can identify whether and how patterns of integration differ for “resolved” research questions compared to “open” questions. To do this, we compute community detection solutions and the correspondence analyses for the collapsed complete corpus (i.e., including all papers within a single analytic corpus), and separately over a series of moving windows that capture relevant “epistemic periods.” These moving windows are labeled by the year at the end of the window and extend backwards for 4 years, which represents the median citation age within this corpus; “Citation age” is the difference (in years) between the date of the citing paper's publication and the year of publication for each of its cited references [38].

## Results

### Networks in the Complete Corpus

First, we present the bibliographic coupling based communities identified for the complete 20-year window collapsed into a single network. Fig. 1 visualizes the community identifications for the complete network (Panel A), and separately for *AIDS* and *JAIDS* (Panels B and C, respectively). The network is clustered into distinct communities (modularity = 0.469), and is dominated by three primary communities (colored red, blue and yellow respectively), with several smaller communities that are peripheral to one of those 3 (6, colored orange, is peripherally connected to 3) or two of those larger communities (4, magenta, and 5, green, are peripheral to 1–2 and 2–3, respectively). As of 1999, both journals introduced article classifications of “Basic,” “Clinical” or “Social and Epidemiological” Sciences, which were applied to the vast majority of subsequently, published articles. The correspondence between the three largest bibliographic coupling network communities and these broad “discipline” like labels is pronounced (presented in Panel D) - with each community dominated by one such label (as marked by its overrepresentation and the substantial underrepresentation of both of the others 1∼Clinical, 2∼Basic, 3∼Social/Epidemiological). The identified discipline-based arrangement of communities is not dependent on which community solution is used. A 3-community solution was also identified which only exacerbates this pattern. Similarly, solutions with larger numbers of communities were nested within those presented, i.e., making finer divisions within, not bridging across the discipline-based communities. The emergent communities based on citation overlaps provide initial indication of the persistence of disciplinary boundaries based on the broad categorizations—basic, clinical, and social/epidemiological scientific—within this cross-sectional view. A dynamic approach that considers topic consolidation complicates this initial overview.

Next we ask how these observed communities account for primary drivers of the modularity between HIV/AIDS research areas. The article labels mentioned above hint at some of those bases (i.e., somewhat determined by a “disciplinary” orientation), but to formalize this further, we examine how readily the bibliographic coupling community structure corresponds with the 30 identified topics that summarize the content of HIV/AIDS research (see S2 Figure for more information on topic labeling). Seventeen topics were comparatively “consolidated” (i.e., highly represented in only 1 community), which is consistent with an interdisciplinary approach (e.g., drug metabolism is consolidated in Cluster 1 – the red cluster in Fig. 1 that is more associated with clinical research, while vaccine development is consolidated in Cluster 2/blue/basic science; for a complete list of the consolidated topics, see S3 Figure).

**Figure 2:**
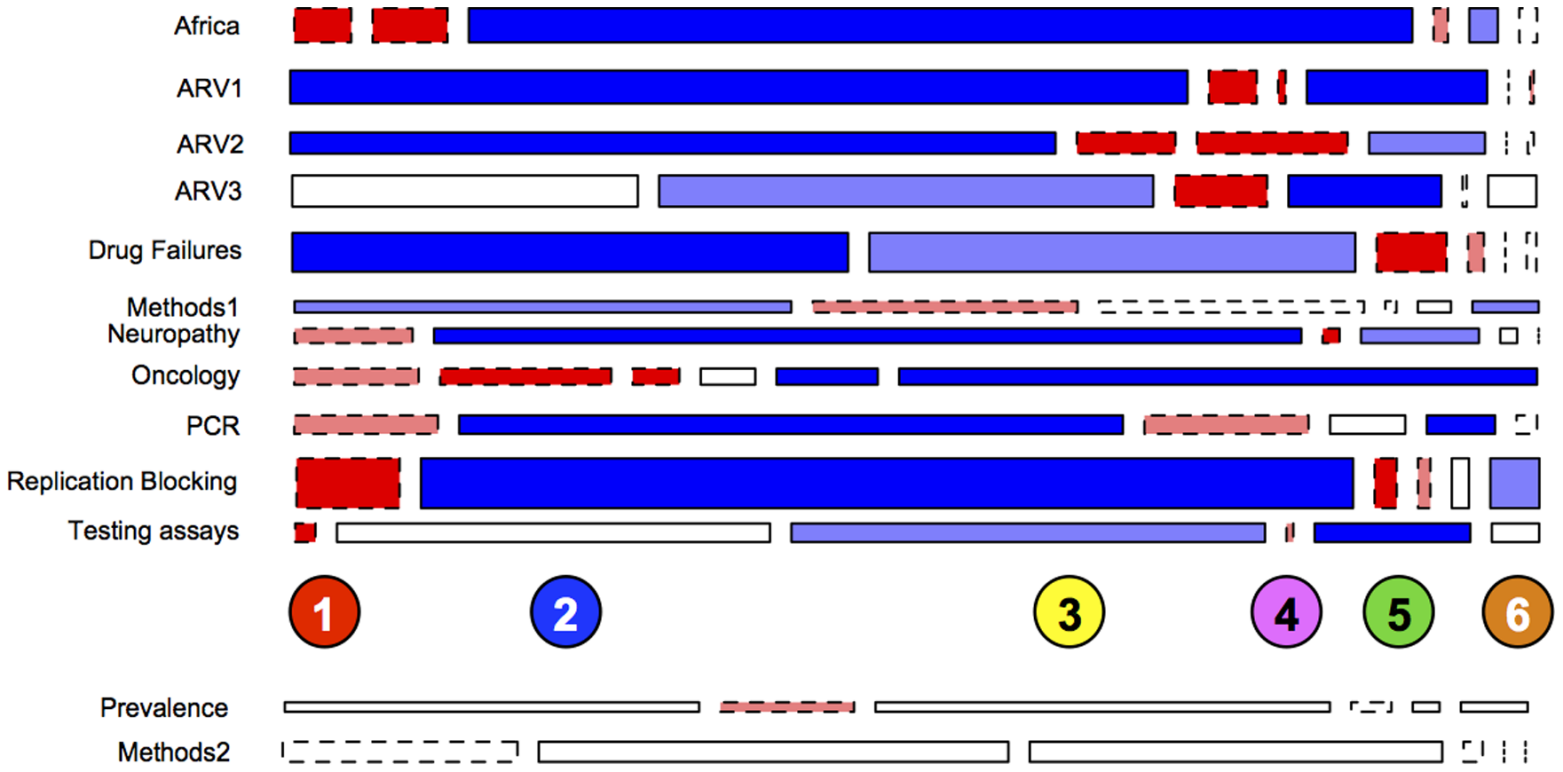
Community-Topic (lack of) Correspondence. This mosaic plot shows those topics that are overrepresented present in more than one network community (top 11), or are not consolidated in any community (bottom 2). The topics are derived via LDA (see Supplementary Information) and the communities are those represented in Fig. 1. Color represents over (blue) and under (red) representation of a topic in a given community according to permutation-based residuals.

Fig. 2 presents a mosaic plot representing correspondence for those 13 topics that are spread over more than 1 community (see S3 Figure for the correspondence of all 30 topics). For example, “ARV3” is a topic about toxicities in clinical trials for antiretrovirals (ARV), which is significantly represented in clusters 2 (blue) and 4 (magenta), and “ARV2,” a topic about ARV treatment adherence, which is present in 1 (red) and 4. This split of single topics across multiple non-overlapping communities thus indicates those topics potentially least coordinated across disciplinary boundaries and, thus, characterized more by multidisciplinarity. The two topics that are evenly distributed across most/all communities provide a meaningful null-result check on the questions here – i.e., by identifying topics that are universally salient (e.g., “Methods 2” which is comprised of language describing measurement and research methods).

### The Evolution of Research Communities & Topics

**Figure 3:**
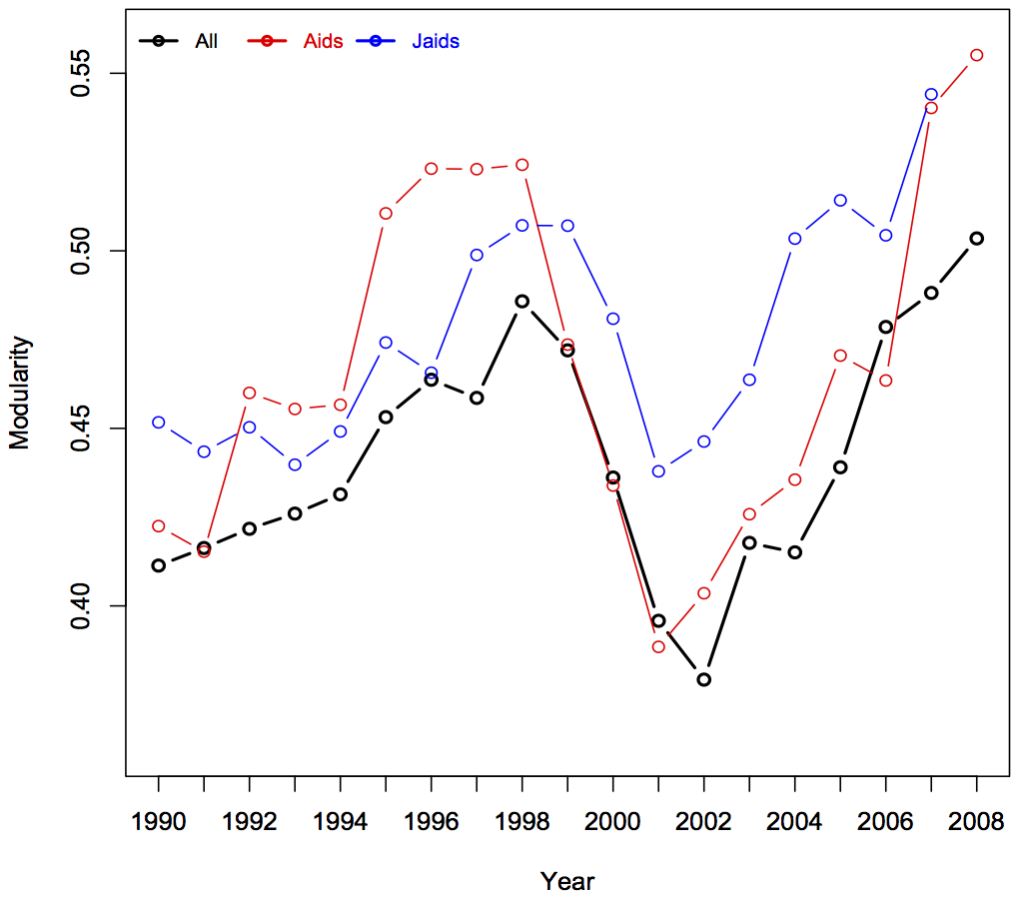
Temporal change in modularity, 1988–2008. Constructed networks comprise all articles published in a 4-year moving window (with labeled year indicating the ending year of that window). For each temporal slice, community detection is applied, and the summary modularity index is presented. The 1998 dip follows the introduction of “discipline” like labels for on all published articles.

It is potentially problematic to consider two decades of HIV/AIDS research as a single corpus. The field has advanced rapidly since these journals were founded in 1988/9 and clustering may have evolved across the observed period. Fig. 3 shows how the bibliographic coupling network's modularity changes across the observed period. Furthermore, this evolution may help to identify temporal patterns that are associated with consensus regarding resolved and/or open questions in the HIV/AIDS research field.

The first noteworthy pattern in Fig. 3 is the general trend of increasing modularity – representing higher segregation of research communities at the end of the period than the beginning. Second, this general pattern is abruptly interrupted with a sharp decrease in both journals following the 1999 introduction of discipline-like labels. This raises an important point about modularity maximization. It is simultaneously capturing two dimensions - the number of communities in the network and the degree to which those communities account for the tie-structure within/between them. The substantial dip following 1999 is driven more by a reduction in the number of salient communities, not a decrease in how segmentation exists between those communities. Third, across most of the window, modularity scores in *AIDS* and *JAIDS* are closely aligned, with changes in *JAIDS* lagging behind those in *AIDS* for roughly the first half of the period, but happening more simultaneously for the latter half.

**Figure 4:**
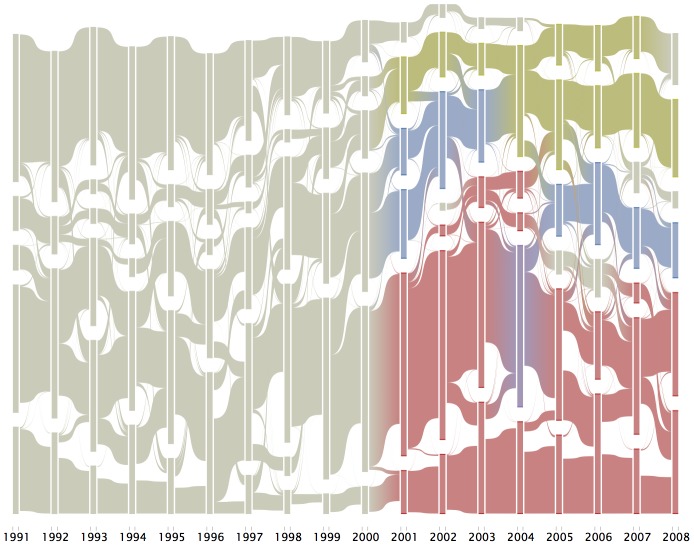
Alluvial Flow Diagram w/“Discipline” Like Labels. This figure presents the evolution of clusters within 5-year moving windows (reduced to include only clusters containing at least 50 papers). The color corresponds to clusters in which the broad “discipline”-like labels are ***over***-represented in a given community (yellow = Social/Epidemiological, blue = Basic, red = Clinical).

Moving to how the bibliographic coupling aligns with the substantive content of the field over time, Fig. 4 shows the temporal evolution of the clusters across 5-year moving windows, overlaid with the correspondence between those clusters and the broad “discipline”-like labels. In any given labeled year, the diagram presents the bibliographic clustering identified communities (bars) for the moving window ending in that year. Between each year, the “flows” between bars indicate the re-arrangement of clusters across the period, with some clusters emerging from the merger of others (see bottom cluster in 2008), others splitting into separate clusters (see the second cluster from the bottom in 2007 splitting across 2 in 2008, and still others remaining relatively consistently comprised (see the top cluster in 2007–08). This diagram allows us to see how the (re-) arrangement of communities progressed through time.

Additionally, by overlaying these changes with the “discipline” like labels from above (which is represented by colors beginning in 2001), we can see what accounts for the structure and dynamics of the changing clustering patterns (see S4 and S5 Figures for the corresponding moving window mosaic plots for broad “discipline” like labels and topics, respectively). Much of the rearrangement between clusters after the implementation of these labels happened within (rather than across) those broad categories (see the rearrangement among the various red clusters), with one notable exception. In the early 2000 s, clusters dominated by “Basic Science” join with and become marked primarily by “Social/Epidemiological Science” (see the transition from blue to yellow near the top of the figure). Then in the mid-/late- 2000 s, new clusters dominated by “Basic Science” emerge from small parts of clusters driven separately by “Clinical” and “Social/Epidemiological” sciences. The dominant pattern in the latter period however is the relative consistency of the clusters that are predominantly Social/Epidemiologically oriented (top/yellow) and those that are predominantly Clinical oriented (bottom/red).

## Discussion and Implications

The high segmentation within the field of HIV/AIDS research is not surprising. In fact, the early period of relative interdisciplinary consolidation is an uncommon pattern among scientific fields. The more important question, therefore, is to identify the primary drivers of the identified community structure and how it evolves over time. The approach presented here however suggests two potential problems with considering the general question of how interdisciplinary HIV/AIDS research is as a field. First, the patterns change substantially over time, and second, the patterns tending more towards multi- or inter- disciplinary integration also differ substantially by topics.

Broadly, what we found in the dynamics of the field as a whole is one that progressed from more interdisciplinary clustering early in the period—highly consolidated, with researchers bridging across disciplinary boundaries to interact around topical themes—to more multidisciplinary clustering later in the period—where researchers from a variety of disciplines are contributing to the field, but are doing so in a way that engages literatures with others from their (cognate) disciplines, regardless of the topic.

The differences in topics' distributions across these clusters are also informative. Some topics retained a relatively consistent pattern over the evolving clustering structure (e.g., vaccine development has remained a relatively consolidated topic throughout, largely within a cluster dominated by basic science research), others remained within a single consolidated cluster, but the disciplinary composition of that cluster changed through time (e.g., the cluster containing research on the HIV-testing assays evolved from being aligned with basic science early in the period to being predominantly social/epidemiological science later in the period - a transition likely consistent with a move from development to use of such tests). Others became more consolidated over time (e.g., molecular strategies for replication blocking of the HIV virus), or remained distributed over multiple communities (e.g., research on treatment failures, especially deriving from developed drug resistance).

While the description above has largely focused on field-level organizational dynamics, the reality is that these patterns are the result of individual- (or research team) level research processes. Those topics marked by consolidation suggest areas where research content drives the literature(s) upon which authors draw in formulating ideas and developing projects. For others (e.g., ARV adherence and drug resistance), it appears that disciplinary boundaries are more likely to shape knowledge production - potentially missing ideas that develop on the same topic, but within another cluster dominated by other disciplines. This leads to the potential of missing key ideas that develop in other (disciplinary) portions of the literature. In sum, the general pattern within HIV/AIDS research is one increasingly marked by disciplinary segmentation rather than integration, while particular topics vary considerably in how closely they adhere to that overall pattern.

The trajectory of interdisciplinary research, such as found in the HIV/AIDS field, is not predetermined. We cannot know from the outset whether a knowledge project will grow more interdisciplinary or will shrink back into disciplinary boundaries. This ambiguity is even more pronounced when considering research fields with heavy investments as various key players may have incentives to prevent or expand integration. The models employed here provide a means for examining the multidimensional and dynamic processes that enable evaluation of progress within interdisciplinary frameworks.

## Supporting Information

S1 Figure **Perplexity Scores by Number of Topics.** This figure presents the optimization information for the number of topics identified within the corpus.
(TIFF)

S2 Figure **Topic Label Contributions by Proportional vs. Top Topic Assignment.** This figure compares the contribution to the overall topic distribution of each of the 30 identified topics. The comparison is between assigning each paper proportionally to the complete set of topics it is identified with versus assigning each paper only to the *single* topic it is most closely identified with.
(TIFF)

S3 Figure **Complete Correspondence between Clusters and Topics.** This figure presents the correspondence analysis for all 30 topics and all 6 clusters. It adds the information for the 17 consolidated topics that are excluded from Fig. 2 in the main text.
(TIFF)

S4 Figure **Evolution of Relationship between Clusters and Discipline-Like Labels.** This figure provides the correspondence between the identified clusters and the broad discipline-like labels separately for 5-year moving windows – the dynamic version of Fig. 1's mosaic plot.
(TIFF)

S5 Figure **Evolution of Relationship between Clusters and Topics.** This figure provides the correspondence between the identified clusters and identified topics separately for 5-year moving windows – the dynamic version of the mosaic plots in Fig. 2 and S3 Figure.
(PDF)

S1 Table **Topic Labels and Brief Descriptions.** This table presents the full list of identified topic labels, and briefly describes the content included in each of those topics.
(PDF)

S1 Text **Description of Supplementary Analyses.** This section provides description of the methods for several ancillary analyses that support the details presented in the manuscript text.
(PDF)
