# Supplementary figures and images for: Mapping Interdisciplinary Fields: Efficiencies, Gaps and Redundancies in HIV/AIDS Research
Source: PLoS One. 2014 Dec 15;9(12):e115092. doi: 10.1371/journal.pone.0115092 (PMC4266665; doi:10.1371/journal.pone.0115092)

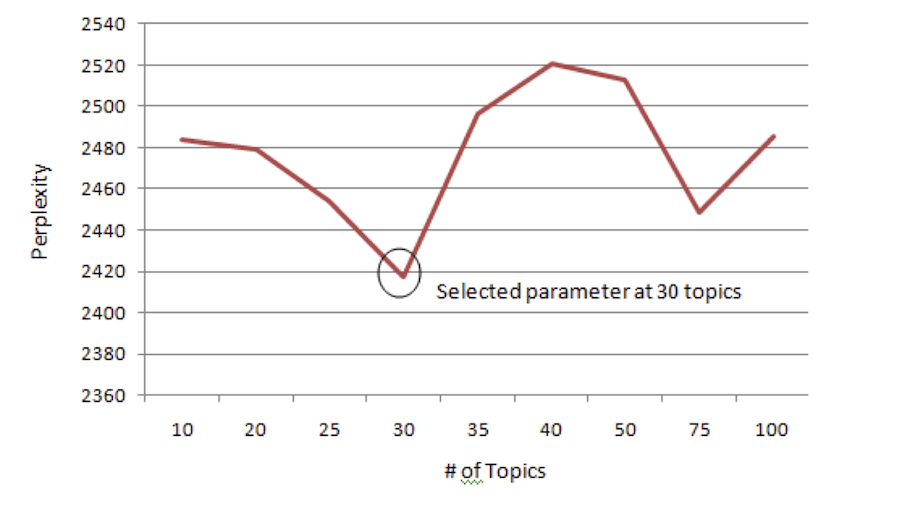

Supplement: S1 Figure — Perplexity Scores by Number of Topics. This figure presents the optimization information for the number of topics identified within the corpus. (TIFF) [file pone.0115092.s001.tiff]

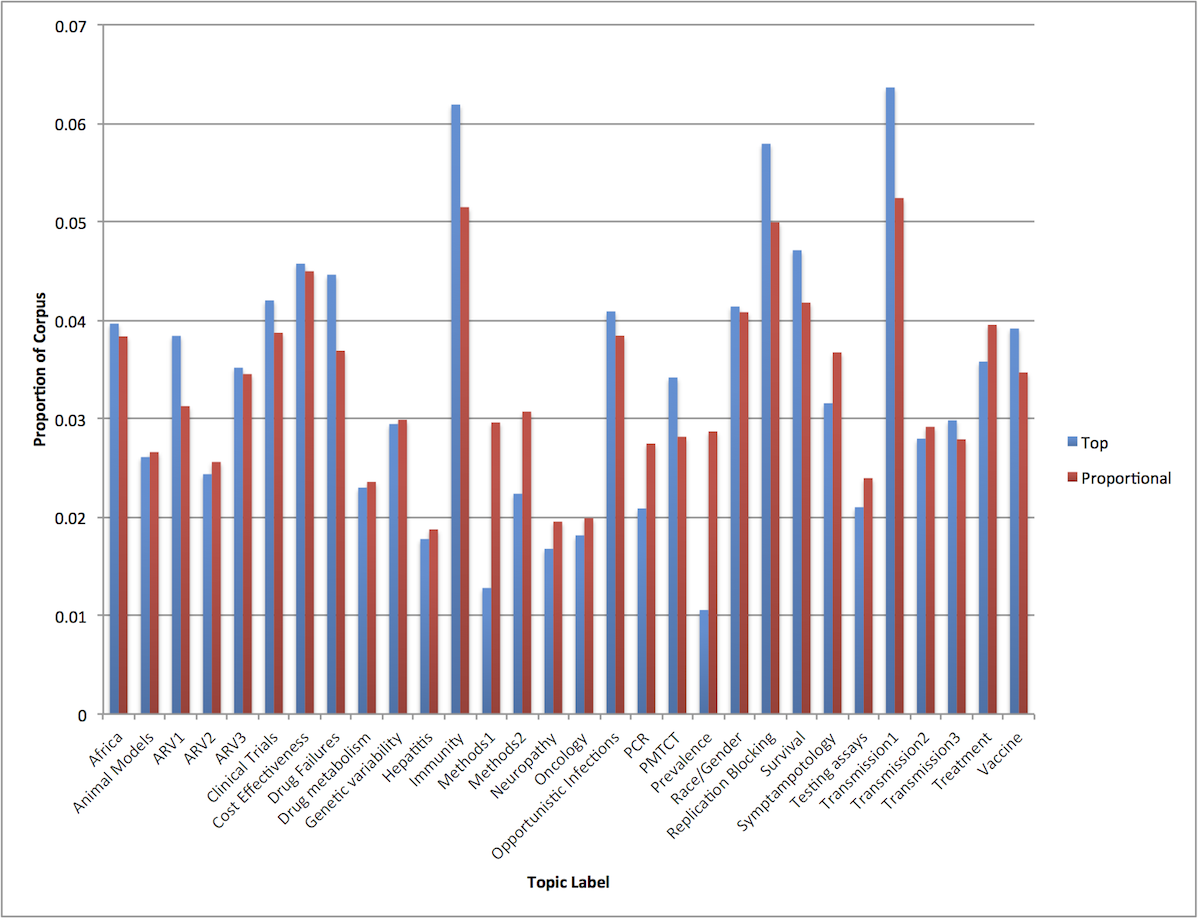

Supplement: S2 Figure — Topic Label Contributions by Proportional vs. Top Topic Assignment. This figure compares the contribution to the overall topic distribution of each of the 30 identified topics. The comparison is between assigning each paper proportionally to the complete set of topics it is identified with versus assigning each paper only to the single topic it is most closely identified with. (TIFF) [file pone.0115092.s002.tiff]

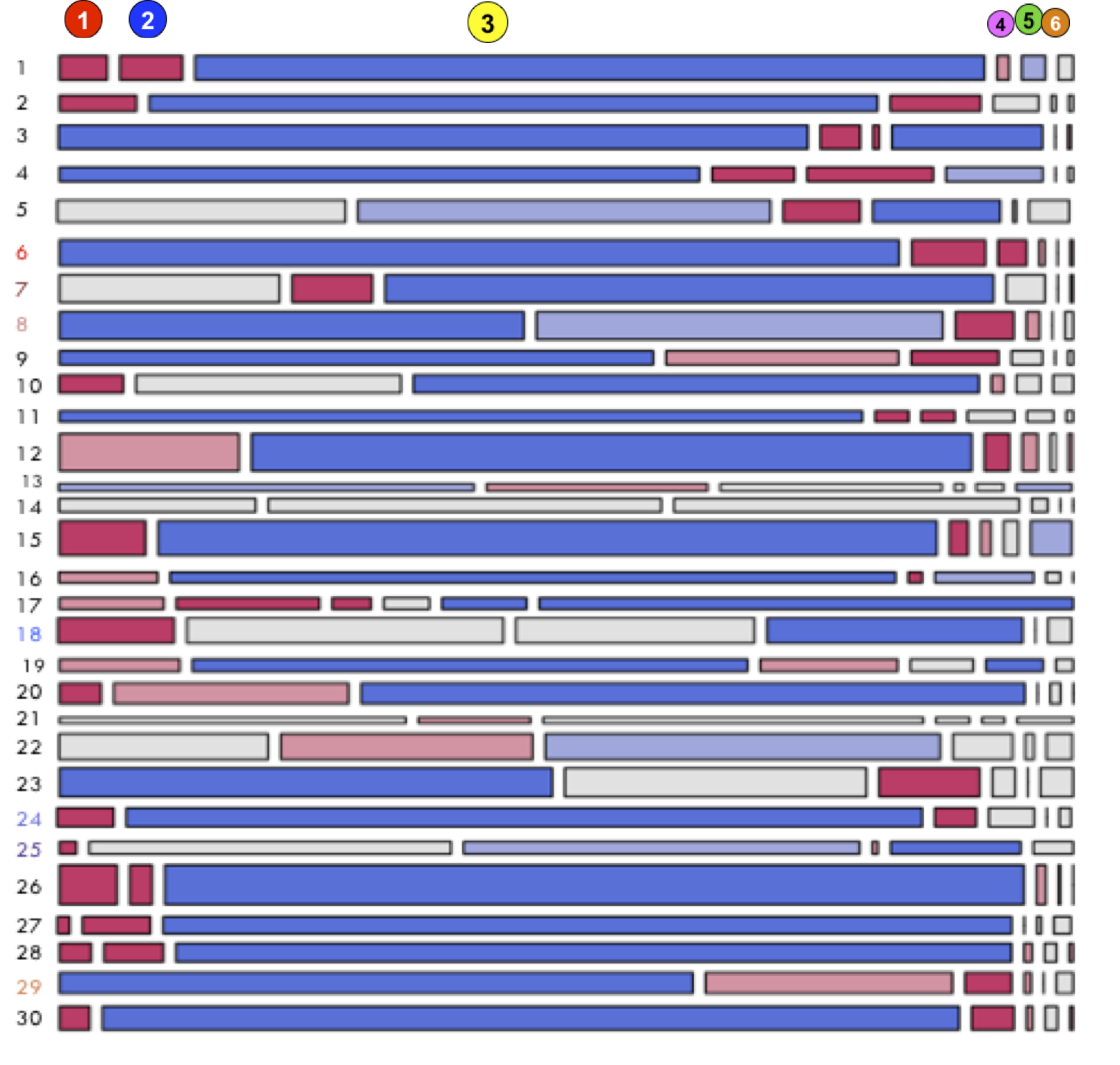

Supplement: S3 Figure — Complete Correspondence between Clusters and Topics. This figure presents the correspondence analysis for all 30 topics and all 6 clusters. It adds the information for the 17 consolidated topics that are excluded from Fig. 2 in the main text. (TIFF) [file pone.0115092.s003.tiff]

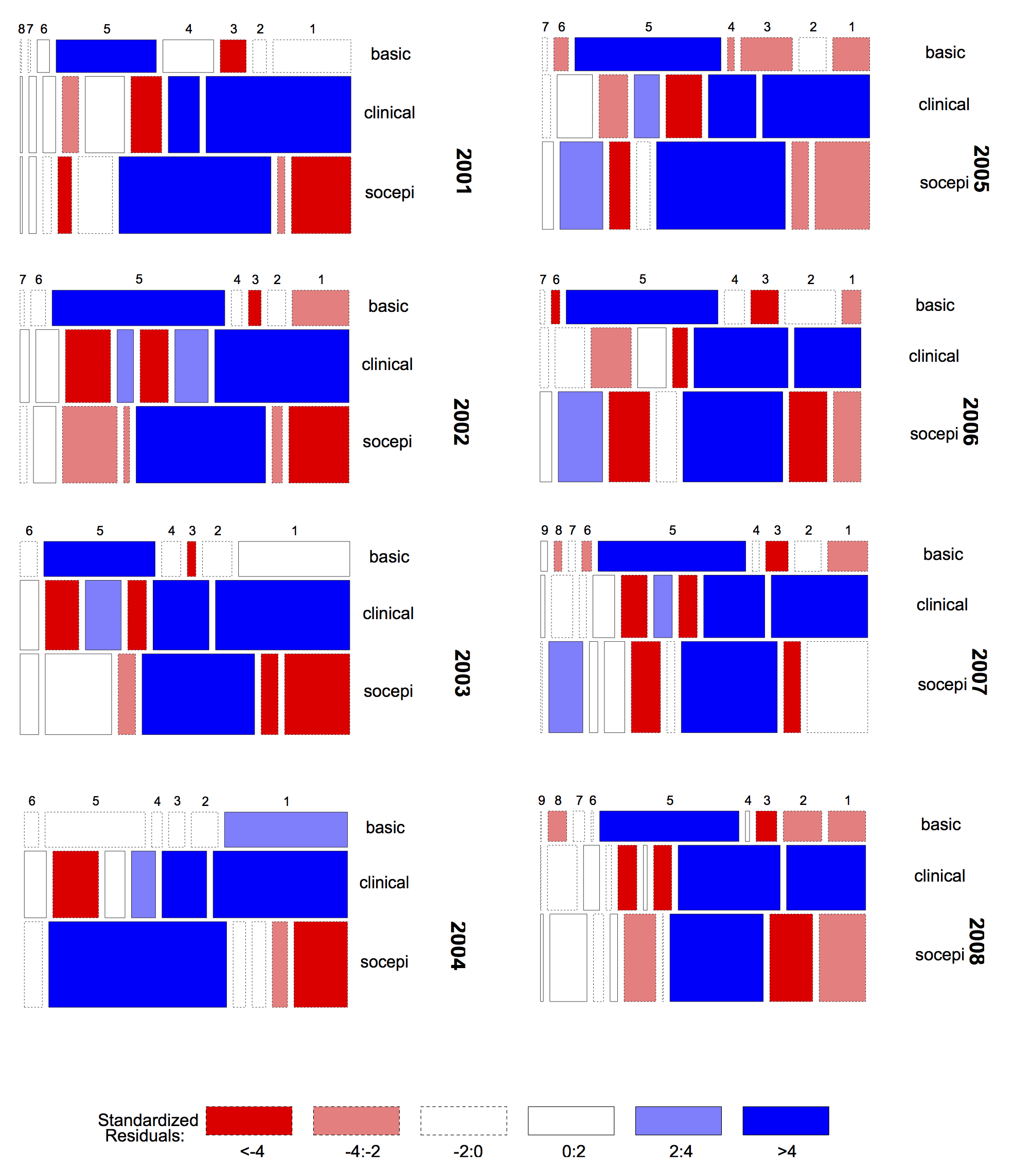

Supplement: S4 Figure — Evolution of Relationship between Clusters and Discipline-Like Labels. This figure provides the correspondence between the identified clusters and the broad discipline-like labels separately for 5-year moving windows – the dynamic version of Fig. 1's mosaic plot. (TIFF) [file pone.0115092.s004.tiff]
