# Supplementary material for: Mapping Interdisciplinary Fields: Efficiencies, Gaps and Redundancies in HIV/AIDS Research
Source: PLoS One. 2014 Dec 15;9(12):e115092. doi: 10.1371/journal.pone.0115092 (PMC4266665; doi:10.1371/journal.pone.0115092)
Supplement: S5 Figure — Evolution of Relationship between Clusters and Topics. This figure provides the correspondence between the identified clusters and identified topics separately for 5-year moving windows – the dynamic version of the mosaic plots in Fig. 2 and S3 Figure. (PDF) [file pone.0115092.s005.pdf]

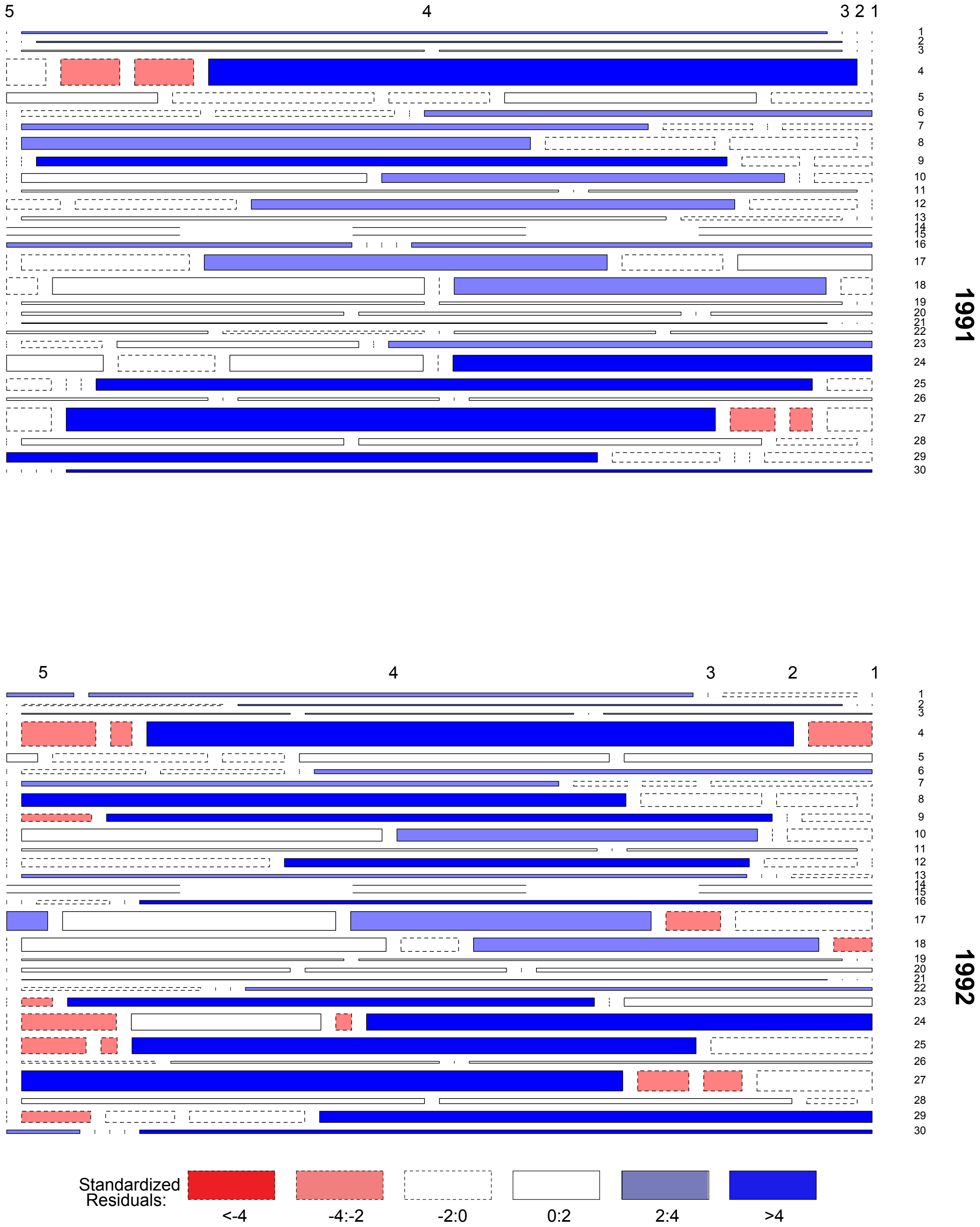

Topic Key:

1. Drug Metabolism
2. ARV1
3. Hepatitis
4. Replication Blocking
5. Animal Models
6. Africa
7. Methods2
8. ARV3
9. Tranmission3
10. Race & Gender
11. Drug Failures
12. Neuropathy
13. Survival
14. Methods1
15. Clinical Trials
16. PMTCT
17. Opportunistic Infections
18. Immunity
19. ARV2
20. Cost Effectiveness
21. Treatment
22. Genetic Variability
23. Transmission1
24. Testing Assays
25. Vaccine
26. Prevalence
27. Symptomatology
28. Oncology
29. PCR
30. Transmission2

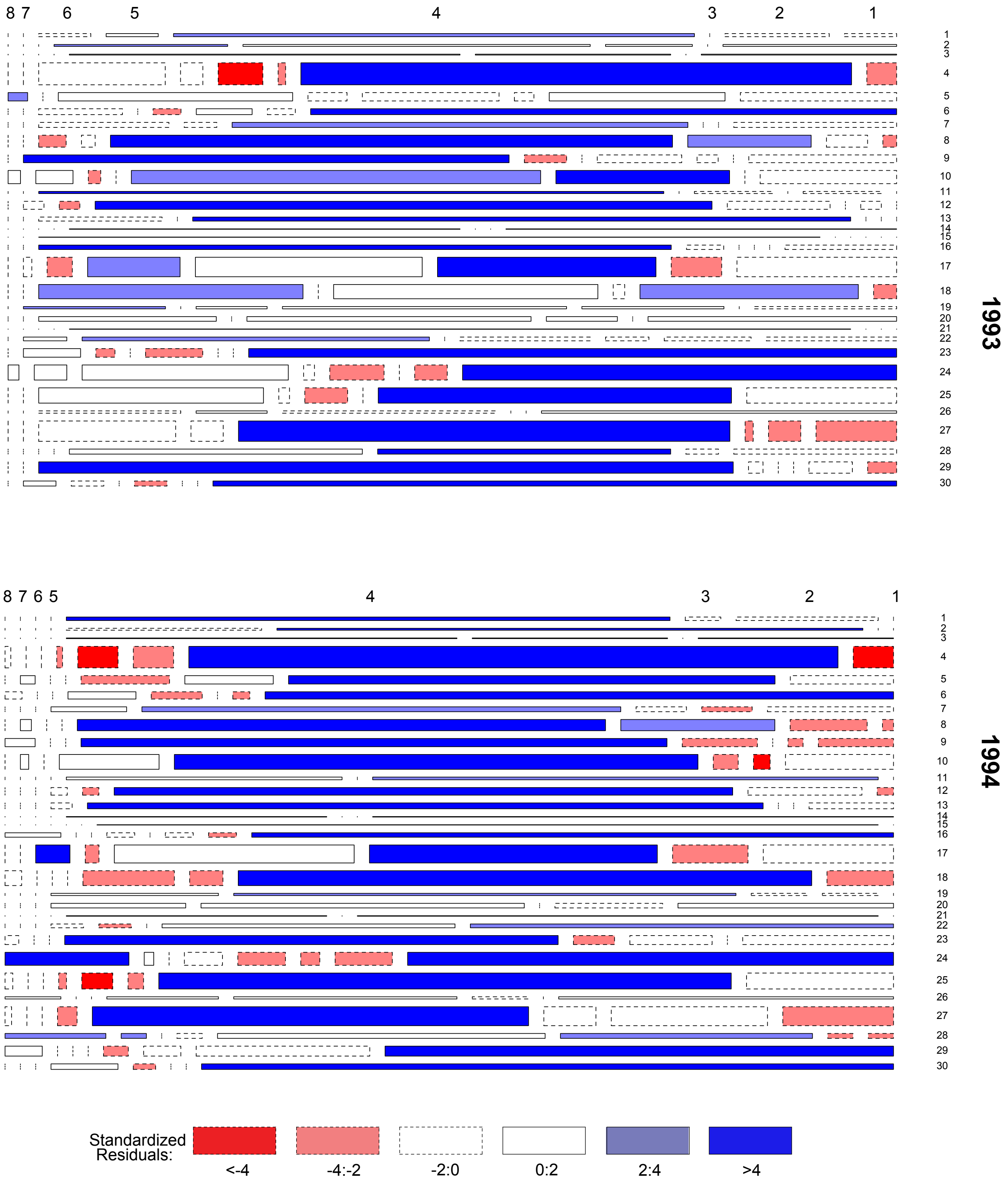

Topic Key:

1. Drug Metabolism
2. ARV1
3. Hepatitis
4. Replication Blocking
5. Animal Models
6. Africa
7. Methods2
8. ARV3
9. Tranmission3
10. Race & Gender
11. Drug Failures
12. Neuropathy
13. Survival
14. Methods1
15. Clinical Trials
16. PMTCT
17. Opportunistic Infections
18. Immunity
19. ARV2
20. Cost Effectiveness
21. Treatment
22. Genetic Variability
23. Transmission1
24. Testing Assays
25. Vaccine
26. Prevalence
27. Symptomatology
28. Oncology
29. PCR
30. Transmission2

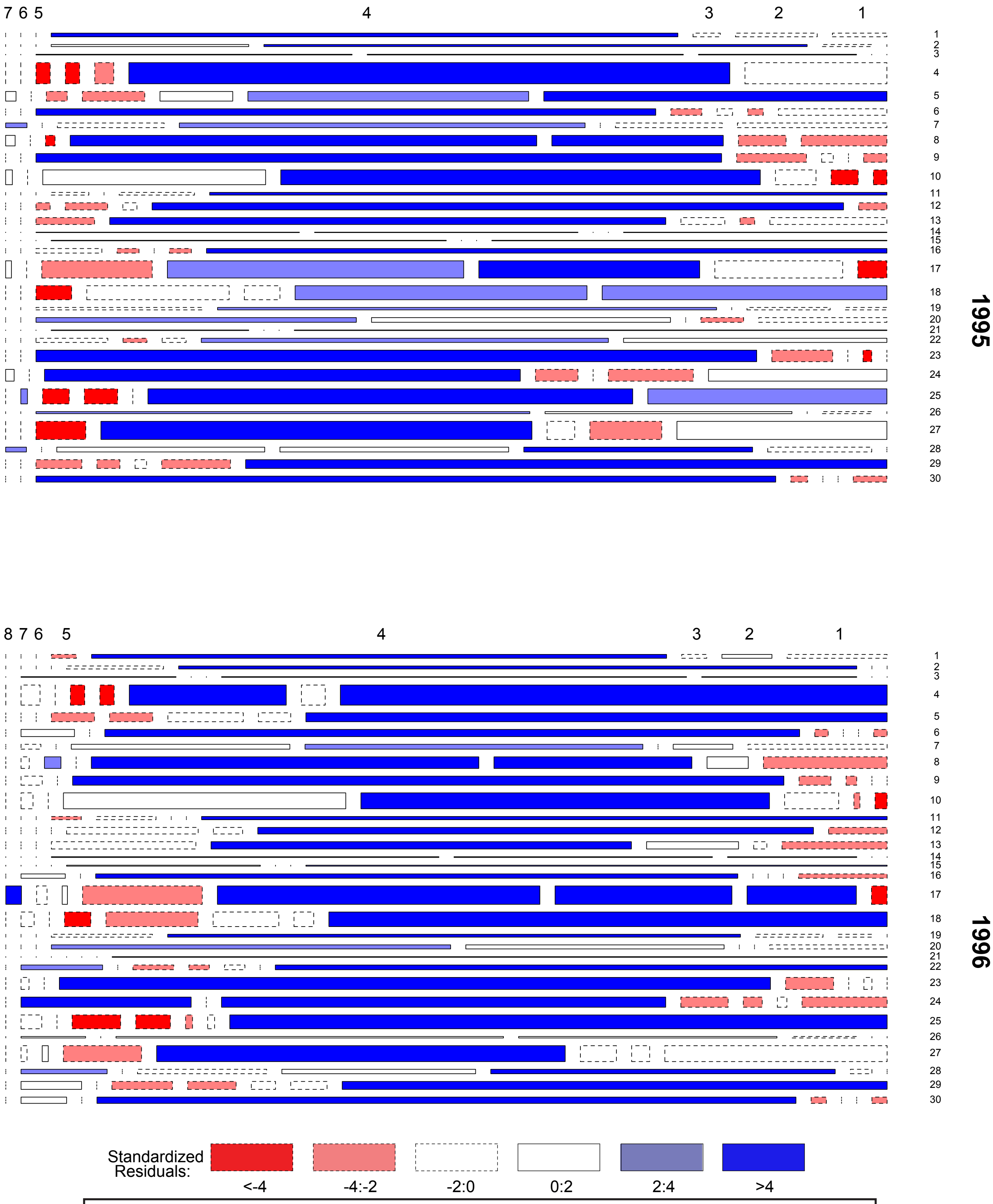

Topic Key:

1. Drug Metabolism
2. ARV1
3. Hepatitis
4. Replication Blocking
5. Animal Models
6. Africa
7. Methods2
8. ARV3
9. Tranmission3
10. Race & Gender
11. Drug Failures
12. Neuropathy
13. Survival
14. Methods1
15. Clinical Trials
16. PMTCT
17. Opportunistic Infections
18. Immunity
19. ARV2
20. Cost Effectiveness
21. Treatment
22. Genetic Variability
23. Transmission1
24. Testing Assays
25. Vaccine
26. Prevalence
27. Symptomatology
28. Oncology
29. PCR
30. Transmission2

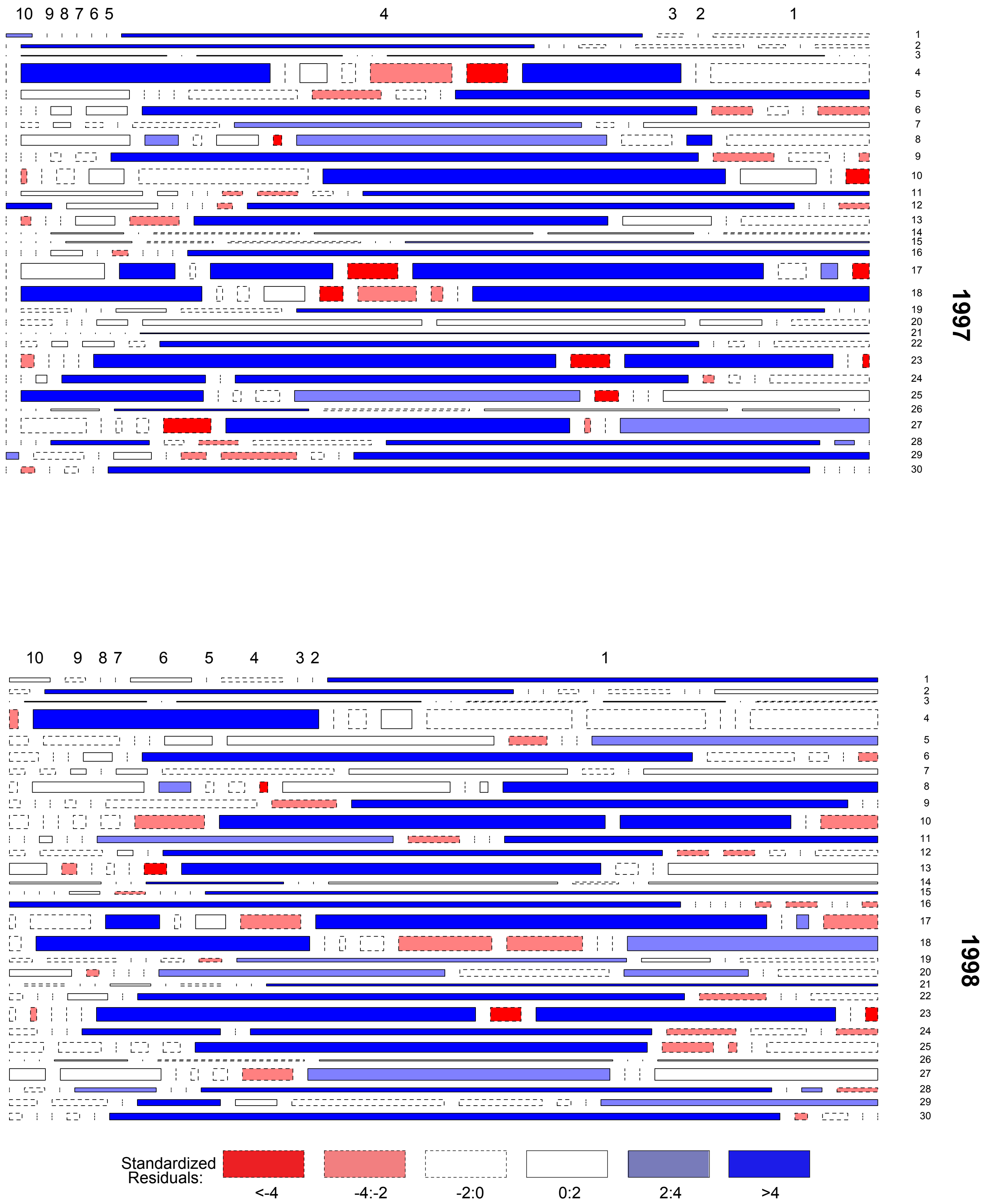

Topic Key:

1. Drug Metabolism
2. ARV1
3. Hepatitis
4. Replication Blocking
5. Animal Models
6. Africa
7. Methods2
8. ARV3
9. Tranmission3
10. Race & Gender
11. Drug Failures
12. Neuropathy
13. Survival
14. Methods1
15. Clinical Trials
16. PMTCT
17. Opportunistic Infections
18. Immunity
19. ARV2
20. Cost Effectiveness
21. Treatment
22. Genetic Variability
23. Transmission1
24. Testing Assays
25. Vaccine
26. Prevalence
27. Symptomatology
28. Oncology
29. PCR
30. Transmission2

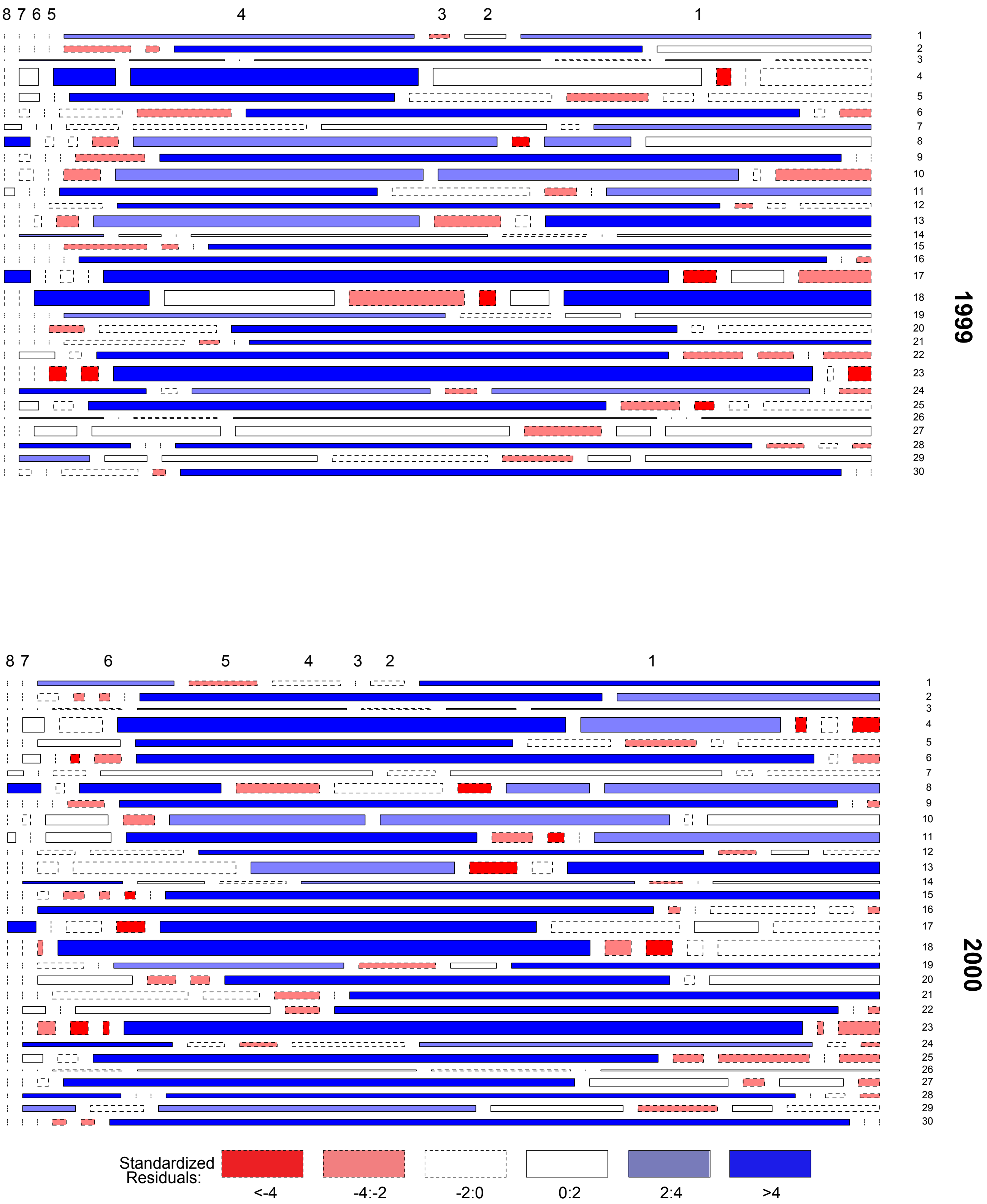

Topic Key:

1. Drug Metabolism
2. ARV1
3. Hepatitis
4. Replication Blocking
5. Animal Models
6. Africa
7. Methods2
8. ARV3
9. Tranmission3
10. Race & Gender
11. Drug Failures
12. Neuropathy
13. Survival
14. Methods1
15. Clinical Trials
16. PMTCT
17. Opportunistic Infections
18. Immunity
19. ARV2
20. Cost Effectiveness
21. Treatment
22. Genetic Variability
23. Transmission1
24. Testing Assays
25. Vaccine
26. Prevalence
27. Symptomatology
28. Oncology
29. PCR
30. Transmission2

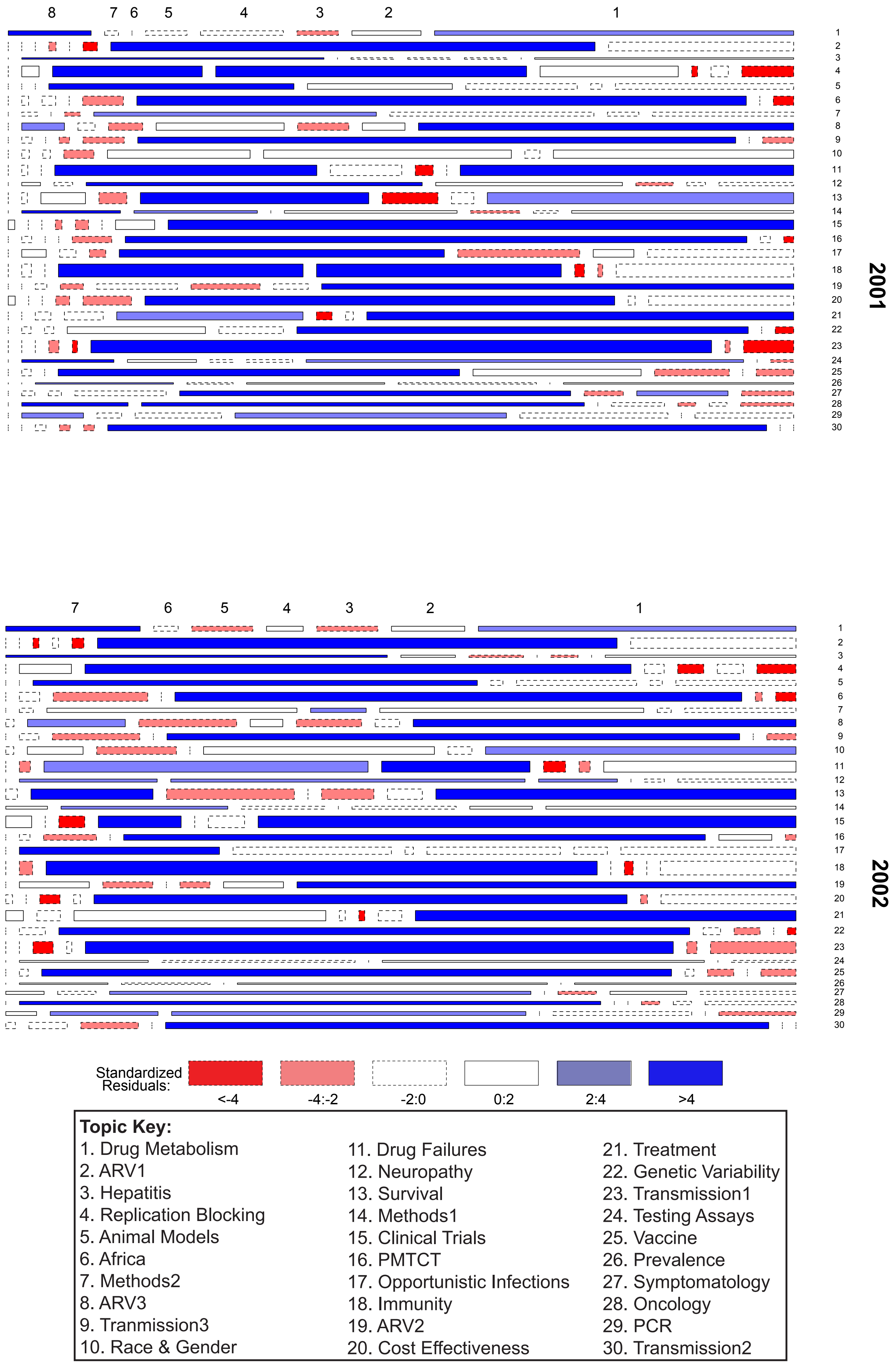
2001
8 7 6 5 4 3 2 1
2002
7 6 5 4 3 2 1
Standardized Residuals:
<-4
-4:-2
-2:0
0:2
2:4
>4
Topic Key:
1. Drug Metabolism
2. ARV1
3. Hepatitis
4. Replication Blocking
5. Animal Models
6. Africa
7. Methods2
8. ARV3
9. Tranmission3
10. Race & Gender
11. Drug Failures
12. Neuropathy
13. Survival
14. Methods1
15. Clinical Trials
16. PMTCT
17. Opportunistic Infections
18. Immunity
19. ARV2
20. Cost Effectiveness
21. Treatment
22. Genetic Variability
23. Transmission1
24. Testing Assays
25. Vaccine
26. Prevalence
27. Symptomatology
28. Oncology
29. PCR
30. Transmission2

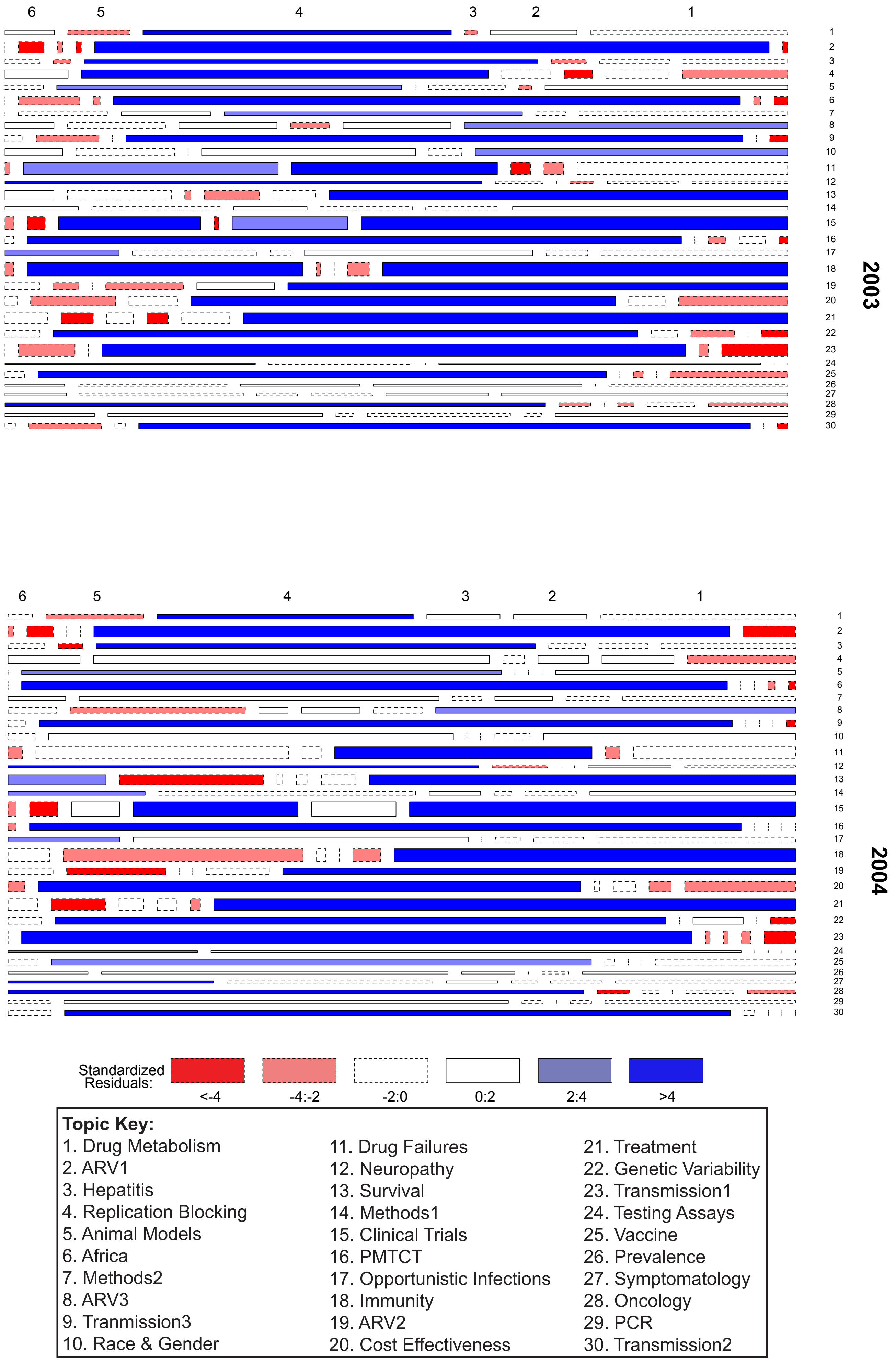

6 5 4 3 2 1
1 2 3 4 5 6 7 8 9 10 11 12 13 14 15 16 17 18 19 20 21 22 23 24 25 26 27 28 29 30
2003
6 5 4 3 2 1
1 2 3 4 5 6 7 8 9 10 11 12 13 14 15 16 17 18 19 20 21 22 23 24 25 26 27 28 29 30
2004
Standardized Residuals:
<-4
-4:-2
-2:0
0:2
2:4
>4
Topic Key:
1. Drug Metabolism
2. ARV1
3. Hepatitis
4. Replication Blocking
5. Animal Models
6. Africa
7. Methods2
8. ARV3
9. Tranmission3
10. Race & Gender
11. Drug Failures
12. Neuropathy
13. Survival
14. Methods1
15. Clinical Trials
16. PMTCT
17. Opportunistic Infections
18. Immunity
19. ARV2
20. Cost Effectiveness
21. Treatment
22. Genetic Variability
23. Transmission1
24. Testing Assays
25. Vaccine
26. Prevalence
27. Symptomatology
28. Oncology
29. PCR
30. Transmission2

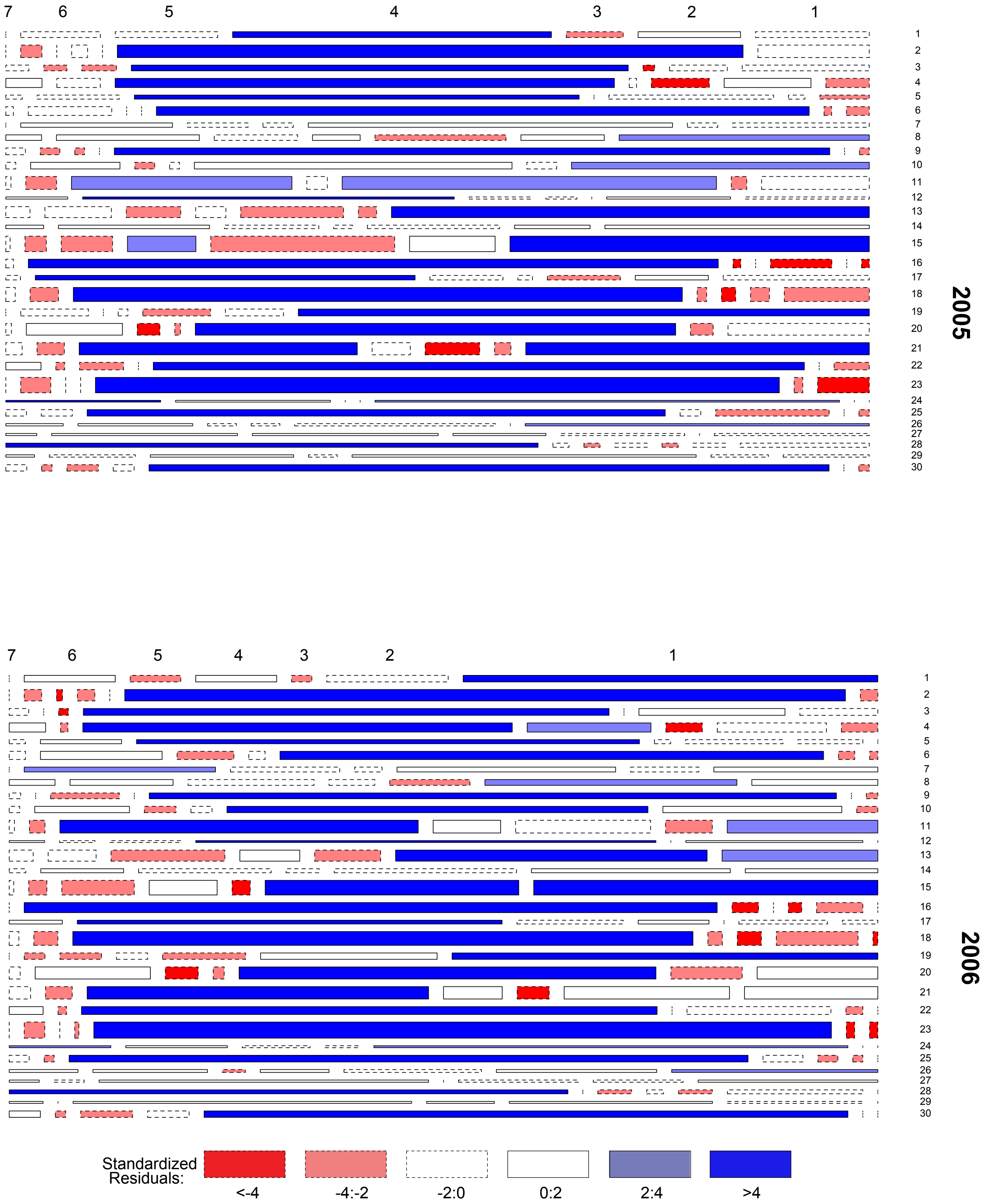

Topic Key:

1. Drug Metabolism
2. ARV1
3. Hepatitis
4. Replication Blocking
5. Animal Models
6. Africa
7. Methods2
8. ARV3
9. Tranmission3
10. Race & Gender
11. Drug Failures
12. Neuropathy
13. Survival
14. Methods1
15. Clinical Trials
16. PMTCT
17. Opportunistic Infections
18. Immunity
19. ARV2
20. Cost Effectiveness
21. Treatment
22. Genetic Variability
23. Transmission1
24. Testing Assays
25. Vaccine
26. Prevalence
27. Symptomatology
28. Oncology
29. PCR
30. Transmission2

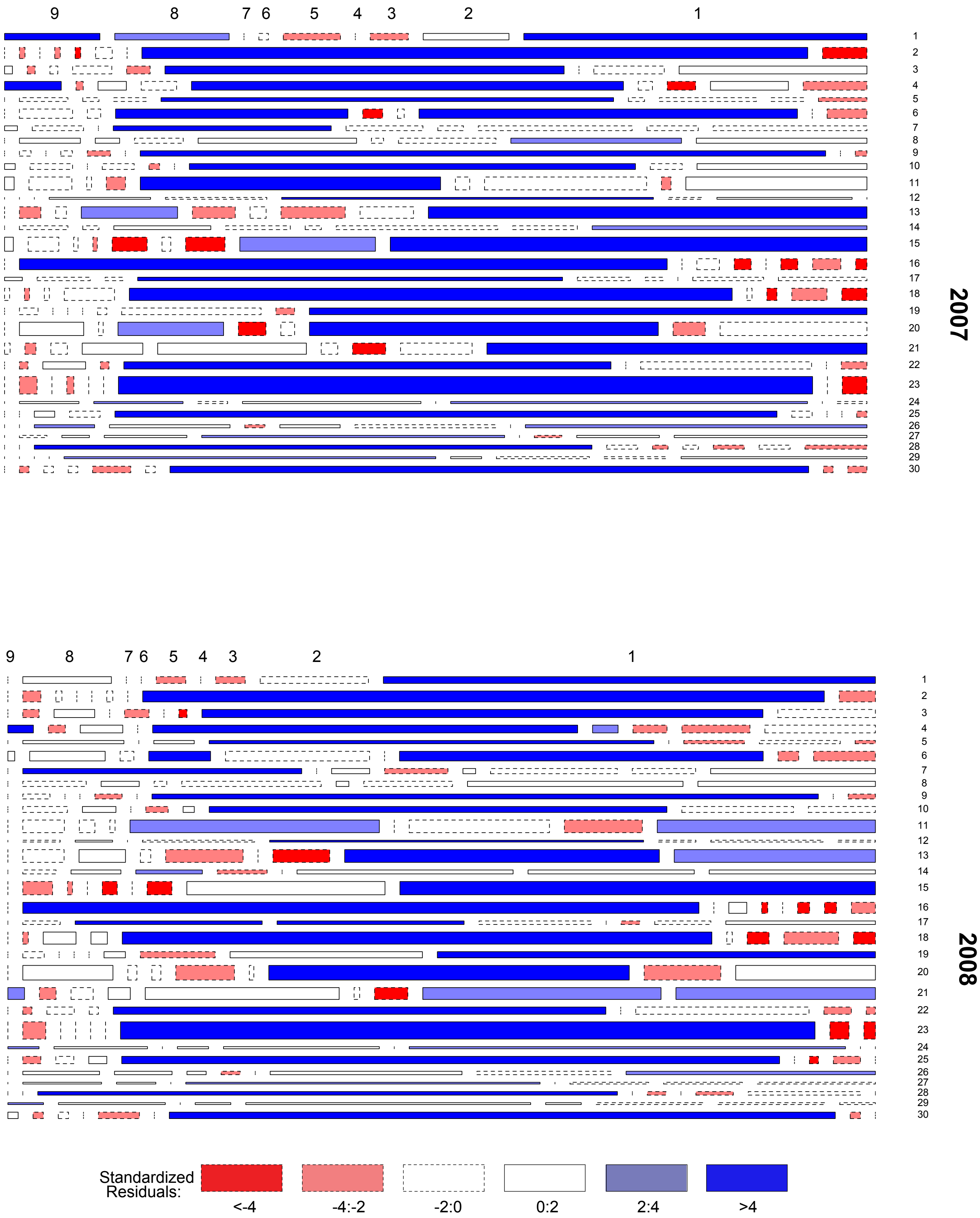

Topic Key:

1. Drug Metabolism
2. ARV1
3. Hepatitis
4. Replication Blocking
5. Animal Models
6. Africa
7. Methods2
8. ARV3
9. Tranmission3
10. Race & Gender
11. Drug Failures
12. Neuropathy
13. Survival
14. Methods1
15. Clinical Trials
16. PMTCT
17. Opportunistic Infections
18. Immunity
19. ARV2
20. Cost Effectiveness
21. Treatment
22. Genetic Variability
23. Transmission1
24. Testing Assays
25. Vaccine
26. Prevalence
27. Symptomatology
28. Oncology
29. PCR
30. Transmission2
