# Supplementary material for: Mapping Interdisciplinary Fields: Efficiencies, Gaps and Redundancies in HIV/AIDS Research
Source: PLoS One. 2014 Dec 15;9(12):e115092. doi: 10.1371/journal.pone.0115092 (PMC4266665; doi:10.1371/journal.pone.0115092)
Supplement: S1 Table — Topic Labels and Brief Descriptions. This table presents the full list of identified topic labels, and briefly describes the content included in each of those topics. (PDF) [file pone.0115092.s006.pdf]

Label	Brief Description
Africa	Socio/behavioral Risk factors of HIV in Africa
Animal Models	Animal Models of HIV/AIDS progression
ARV1	Metabolic complications of antiretroviral treatments
ARV2	Determinants of adherence to antiretroviral drug treatment
ARV3	Toxicities of antiretroviral drugs in clinical trials
Clinical Trials	Clinical testing of anti-HIV drugs
Cost Effectiveness	HIV mitigation program cost effectiveness tradeoffs
Drug Failures	Failure of therapy/drug resistance
Drug metabolism	Metabolism of anti-HIV drugs
Genetic variability	Genetic variability and evolution of HIV worldwide
Hepatitis	Clinical interactions of HIV and viral hepatitis infections
Immunity	Cellular immunity against HIV
Methods1	Lab-focused methodological approaches
Methods2	Quantitative/Statistical methodological approaches
Neuropathy	Neurologic complications of HIV infection
Oncology	Oncologic complications of HIV
Opportunistic Infections	AIDS-related opportunistic infections
PCR	HIV detection and quantification methods
PMTCT	Preventing mother to child (vertical) transmission
Prevalence	HIV/AIDS prevalence estimation & surveillance
Race/Gender	US-based demographic prevalence differences (race/gender)
Replication Blocking	Molecular strategies to block HIV replication
Survival	Survival of people living with HIV/AIDS (PLWHA)
Symptomatology	HIV-specific morbidity / symptomatology
Testing assays	HIV diagnostics
Transmission1	Risk factors associated with sexual transmission
Transmission2	"High risk" sexual transmission associated w/ commercial sex work & STD co-infection
Transmission3	Intravenous drug use -based transmission
Treatment	Efficacy of treatment
Vaccine	Vaccine strategies for HIV
