# Supplementary material for: Mapping Interdisciplinary Fields: Efficiencies, Gaps and Redundancies in HIV/AIDS Research
Source: PLoS One. 2014 Dec 15;9(12):e115092. doi: 10.1371/journal.pone.0115092 (PMC4266665; doi:10.1371/journal.pone.0115092)
Supplement: S1 Text — Description of Supplementary Analyses. This section provides description of the methods for several ancillary analyses that support the details presented in the manuscript text. (PDF) [file pone.0115092.s007.pdf]

Description of Supplementary Analyses for:

Mapping Interdisciplinary Fields: Efficiencies, Gaps & Redundancies in HIV/AIDS Research

Topic Models: Determining the Number & Labeling of Topics

The literature has not converged on a single technique in locating topics and topic modelers in fact appear reluctant to commit to any of the proposed methods. However, several of the developers have suggested that perplexity scores may be the best option in assessing the generalizability of topic models (1). Perplexity compares the success of the solutions on the “held out” text. The lower the perplexity score, the better the model. Blei, Ng, and Jordan (2) describe perplexity as “algebraically equivalent to the inverse of the geometric mean of per word likelihood” (p.1010). Perplexity is a local maximization algorithm. As can be seen in Figure S1, for this corpus, it identifies either 30 or 75 topic solutions.

Once the model identifies the number of topics, it produces several sets of output. The probabilistic assignment of papers to each topic are the data used in the primary analyses presented in the manuscript. In addition to this, the model also provides a list of the top word (stems) associated with each topic. For our models, we provided the top 50 words associated with each topic to 4 separate HIV experts, whose collective training/research/teaching experience covers biology, demography, epidemiology, genetics, clinical medicine, sociology, vaccine development and virology. Each independently coded the topics. Those codings were summarized by the first author then returned to each coder for confirmation. In the two cases where differences arose from the independent codings, focused discussions helped to resolve conflicts. It is important to note that this coding process is only providing interpretation, not computation/generation of the topic models. Table S1 provides the applied labels and short descriptions for each of the identified 30 topics.

These topics are not equally present within each article in the corpus. The topic model results provide a proportional allocation of the probability of each abstract deriving from each of the identified

topics. Figure S2 presents 2 different ways to use this information to allocate each abstract in the corpus to the identified list of topics. First, each paper can be considered as being assigned to a single topic, which we do simply identifying the largest probability for each paper ("Top" topic allocation). The second option considers that most research likely addressed more than one topic at a time and thus assigns abstracts to topics proportionally. As can be seen, the distributions do not differ appreciably depending on which of these allocation strategies we use. In the analyses presented in the manuscript, all topic allocations (and correspondence comparisons) are conducted using the proportional allocation strategy. Results did not differ in any appreciable ways if instead using the top topic allocation approach.

The following figures provide several expanded details of the correspondence comparisons between the identified clustering and subject headings/topics. Figure S3 adds the "consolidated" topics that are excluded from Figure 2 in the manuscript. Figures S4 & S5 provide the same information separately for each of the 5-year moving time slices across the observed window, which provide the basis for several of the interpretations in the discussion section.
